# Changing non-participation in epidemiological studies of older people: evidence from the Cognitive Function and Ageing Study I and II

**DOI:** 10.1093/ageing/afv101

**Published:** 2015-08-20

**Authors:** Lu Gao, Emma Green, Linda E. Barnes, Carol Brayne, Fiona E. Matthews, Louise Robinson, Antony Arthur

**Affiliations:** 1 MRC Biostatistics Unit, Cambridge Institute of Public Health, Cambridge, UK; 2 Department of Public Health and Primary Care, Cambridge Institute of Public Health, Cambridge, UK; 3 Institute of Health and Society, Faculty of Medicine, Newcastle University, Newcastle, UK; 4 School of Health Sciences, University of East Anglia, Norwich, UK

**Keywords:** non-participation, epidemiological study, older people

## Abstract

**Background:** non-participation in epidemiological studies threatens the generalisability of findings.

**Objective:** to investigate the change in non-participation between the Medical Research Council Cognitive Function and Ageing Study (CFAS) I and II.

**Design:** a comparison of two epidemiological studies of older people using identical methods.

**Setting:** three geographical areas of the United Kingdom.

**Subjects:** older people aged 65 years and over.

**Methods:** the two studies were conducted approximately two decades apart between 1989 and 1994 (CFAS I) and between 2008 and 2011 (CFAS II). Random samples were drawn from primary care lists. We compared demographic factors associated with non-participation.

**Results:** non-participation in CFAS II was higher than in CFAS I (45.3 versus 18.3%). After adjustment for confounders, in both CFAS I and CFAS II, women were more likely to decline to take part (CFAS I: odds ratio (OR) 1.3 95% confidence interval (CI) 1.2 to 1.4; CFAS II: 1.1 95% CI 1.1 to 1.2). Deprivation was associated with non-participation in both studies (highest versus lowest Townsend deprivation quintile, CFAS I: OR 1.4 95% CI 1.2 to 1.6; CFAS II: 2.0 95% CI 1.8 to 2.2). Age was not associated with non-participation in either study (CFAS I, *P* = 0.21; CFAS II, *P* = 0.47).

**Conclusions:** non-participation in epidemiological studies of older people has increased substantially in the past two decades and public willingness to take part in studies of this kind would appear to be declining. As communities become more diverse and older people have increasing commitments on their time, new ways to engage prospective participants are urgently needed.

## Introduction

Epidemiological studies of disease prevalence and incidence need to be representative of the populations to which their findings are applied for health service planning and delivery to be efficient and equitable. A high level of non-participation by those invited to take part is likely to be the greatest threat to generalisability. Non-participation bias, where those who take part differ in important ways from those who do not, can lead to uncertainty and error in inferences made from samples to the wider population.

It has been widely reported that participation in epidemiological studies has decreased over time [1, 2–4]. A review of studies published in 2003 found that participation fell between 1970 and 2002 for all types of study design [4]. Evidence from the United States suggests that older adults are less likely to participate in research than younger adults although only 1 in 10 studies attempted to compare those who do and do not participate and only a minority of non-participants are willing to provide a reason for their non-participation [5].

Representative samples of older people are required for the study of population ageing, but factors associated with greater age such as cognitive impairment, frailty and care home residence present potential barriers to study recruitment. Large population studies of older people report variable participation [6–8]. Direct comparisons are problematic given the variation in target sample, extent of prior contact with potential participants and method of approach.

The MRC Cognitive Function and Ageing Studies (CFAS) I and II are multi-centre epidemiological studies of people aged 65 years and over conducted approximately two decades apart using identical recruitment methods in the same geographical areas of England. We aimed to compare non-participation in the two studies, identify demographic factors associated with non-response and examine reasons given for non-participation.

## Methods

Fieldwork for the original CFAS was conducted in six geographical areas of the United Kingdom (Cambridgeshire, Gwynedd, Liverpool, Newcastle, Nottingham and Oxford) between 1989 and 1994. CFAS II was conducted between 2008 and 2011 and restricted to three of the original areas (Cambridgeshire, Newcastle and Nottingham). Analyses reported here are restricted to the three geographical areas involved in both studies (referred to here as CFAS I and CFAS II). Sampling, access and initial approach to potential participants, and recruitment were conducted in an identical manner in CFAS I and CFAS II.

The process of identification and recruitment is illustrated (see Supplementary data, Webfigure S1, available in *Age and Ageing* online). For sampling, CFAS I and CFAS II drew on primary care registration, the most robust method for epidemiological studies conducted in the United Kingdom. Once all general practices were identified within each of the geographical areas, the local Family Health Services Authorities (CFAS I) and Primary Care Trusts (CFAS II) provided a pseudonymised number, gender, general practitioner (GP) surgery and date of birth for all patients registered at these practices aged 65 years and over. This formed the sampling frame from which a random sample was drawn to ultimately secure 2,500 participants within each area (7,500 people in total). Addresses were provided for those randomly selected. For both CFAS I and II, sampling was stratified according to age group with equal numbers aged 65–74 years and aged 75 years and over.

GPs in each of the CFAS areas were contacted about the study. If they agreed to take part, a list of the selected patients was sent to the practice to ensure these patients were still registered and their details were correct. We asked GPs to exclude patients in the final stages of a terminal illness or where there was perceived to be a safety risk to the study interviewer.

Individuals received an invitation letter jointly signed by their GPs and the local principal investigator, a patient information sheet and a photograph of the named interviewer who would visit within 7 days. Potential participants were told that the interview would last ∼90 min. Although it was made clear that their participation was highly valued, there was no offer of financial or material incentives. Upon visiting, the interviewer explained the study giving the participant an opportunity to ask questions about their potential involvement. If the participant agreed, a mutually convenient appointment was arranged to conduct the interview. If those declining to take part were willing to give a reason, then, for CFAS II only, this was recorded and categorised.

If the individual at approach was deemed not to have mental capacity (Mental Capacity Act 2005) or unable to participate due to severe frailty, then a request was made to speak to an informant who could provide information about their health on their behalf. In addition, an informant interview was requested in a stratified random sub-sample of the participants. The interviews included questions about lifestyle, health, activities of daily living, assessment of cognition, use of services and medication. In both studies, participants had the opportunity to express an interest in brain donation following death. In CFAS II, participants were additionally asked to provide a saliva sample creating a research resource that could include genetic (DNA) tests.

Ineligibility for individuals was categorised as: errors within records obtained from the FHSA/PCT; falling outside the study inclusion criteria and not being contactable. Participants included eligible individuals who either consented to and completed an interview or were unable to be interviewed but an informant provided information on their behalf. Non-participation was calculated as the number of non-participants divided by number of eligible contactable individuals. To investigate the relationship between demographic factors and non-participation, we used data available for both participants and non-participants: gender, age at sampling date, postcode level of Townsend deprivation index [9] and care home residence (CFAS II only).

### Data handling and statistical analysis

Data checking, cleaning, encoding and auditing were carried out at the Cambridge centre. Version 2 of the data is used in this paper. Multivariable logistic regression models were fitted to estimate odds ratios for the adjusted associations between non-participation and demographic variables (geographical area, sex, age, Townsend deprivation index) for each study. To test whether associations between non-participation and each of the four demographic variables differed between the two studies, a multivariable logistic regression model was fitted which included four interaction terms. Age at sampling date and Townsend deprivation index were non-normally distributed and therefore treated as discrete variables in the models. People were grouped into 5-year age groups and five groups based on cohort-specific quintiles of Townsend deprivation index. Stata 12.1 under UNIX system was used for data handling, management and analysis.

### Ethical approval

CFAS I and CFAS II interviewing has been given local and multi-centre ethical approval (CFAS I: MREC99/5/22, 05/MRE05/37; CFAS II: 07/MRE05/48).

## Results

**Figure 1.**
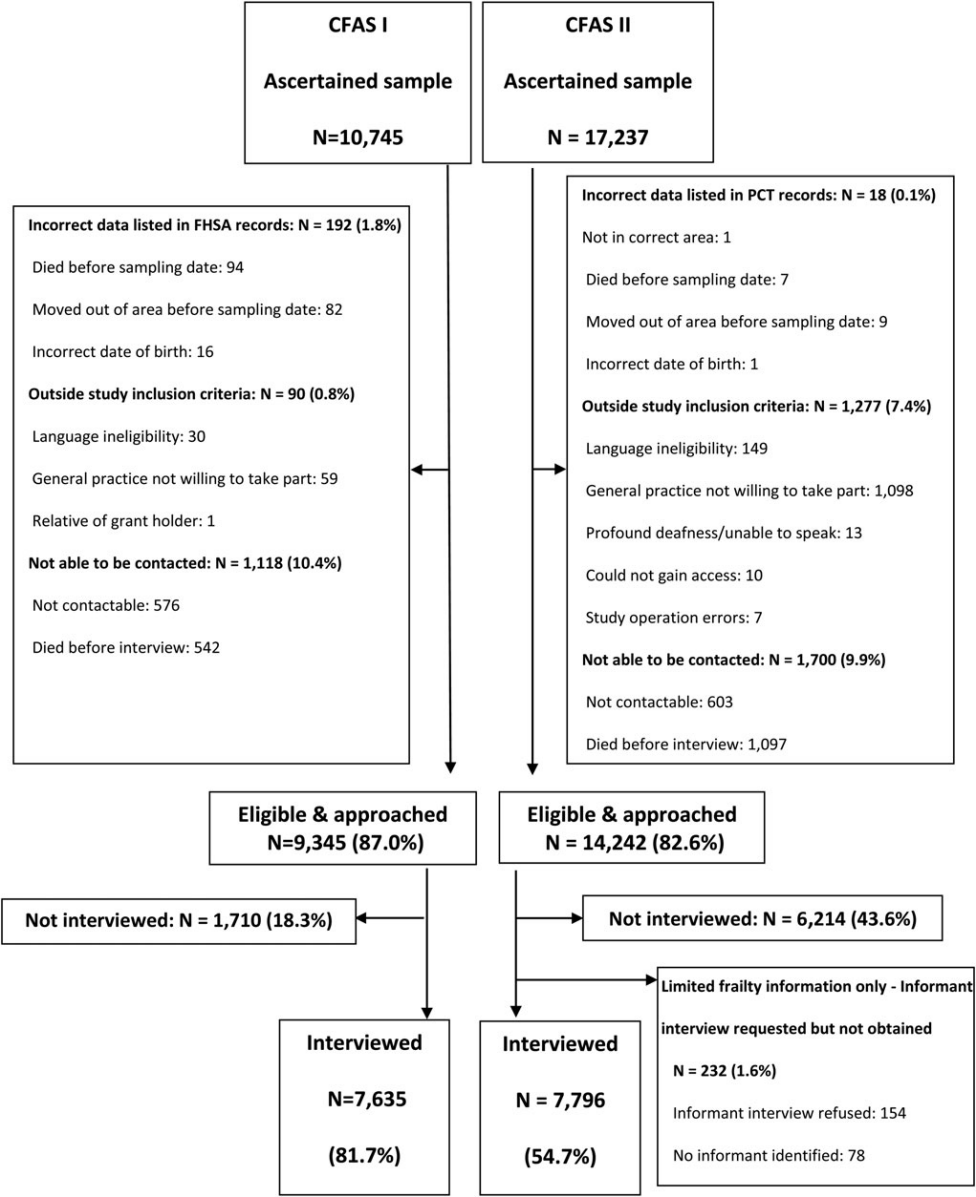
Flow of participants from identification to participation in CFAS I and CFAS II.

Figure 1 reports sampling and participation at each stage of identification and recruitment in the two studies. Inaccuracies in study data were higher in CFAS I, whereas more potential participants were excluded in CFAS II as a result of more general practices being unwilling to help facilitate the study, along with nearly five times as many individuals excluded due to language ineligibility. The proportion of the ascertained sample unable to be contacted was similar in the two studies but was more frequently due to death in CFAS II than that in CFAS I (see Supplementary data, Webtable S1, available in *Age and Ageing* online). Individuals who died before interview were more likely to be older and had higher deprivation scores.

Non-participation from the eligible and approached sample in CFAS II was substantially higher than in CFAS I (45.3 versus 18.3%). In CFAS II, this meant a greater number of potential participants had to be sampled to achieve the target of 2,500 participants in each geographical area.

Table 1 reports differences in the socio-demographic profile of non-participants and participants from the eligible sample, in the two studies. In CFAS II, there were differences in non-participation between geographic areas ranging from 39.5 to 46.5%, but absolute differences in CFAS I were negligible (test for interaction *P* < 0.01). Women were more likely to be non-participants in both studies (CFAS I: OR 1.3 95% CI 1.2 to 1.4; CFAS II: 1.1 95% CI 1.1 to 1.2). We found no evidence of any age difference in non-participation in either study. Living in an area of high deprivation was associated with greater non-participation in both studies though the effect was greater in CFAS II (test for interaction, *P* < 0.01). Compared with people in the lowest quintile of deprivation, people in the highest quintile were those most likely to not participate (CFAS I: OR 1.4 95% CI 1.2 to 1.6; CFAS II: 2.0 95% CI 1.8 to 2.2). In CFAS II, non-participation was lower among care home residents (61/258 23.6% versus 6,153/13,752 44.7%, *P* < 0.001), but data were not available for comparison with CFAS I.

**Table 1.** Distribution of socio-demographics in non-participants and participants, and association between socio-demographics and participation in CFAS I and CFAS II

Characteristics	CFAS I				CFAS II				*P* value**
	Not interviewed, *n* = 1,710 (%)	Interviewed, *n* = 7,635 (%)	Adjusted OR (95% CI)^a^	*P* value	Not interviewed, *n* = 6,214 (%)	Interviewed, *n* = 7,796 (%)	Adjusted OR (95% CI)^a^	*P* value	
Geographical area									
Cambridgeshire	641 (19.8)	2,601 (80.2)	1		1,667 (39.5)	2,558 (60.5)	1		
Newcastle	515 (17.0)	2,522 (83.0)	0.8 (0.7–0.9)	0.01	2,277 (46.5)	2,616 (53.5)	1.3 (1.2–1.5)	<0.01	<0.01
Nottingham	554 (18.1)	2,512 (81.9)	0.9 (0.8–1.0)		2,270 (46.4)	2,622 (53.6)	1.3 (1.2–1.5)		
Sex									
Men	588 (16.2)	3,045 (83.8)	1		2,646 (42.6)	3,550 (57.4)	1		
Women	1,122 (19.6)	4,590 (80.4)	1.3 (1.2–1.4)	<0.01	3,574 (45.7)	4,246 (54.3)	1.1 (1.1–1.2)	<0.01	0.057
Age at sampling date									
<70	410 (19.3)	1,713 (80.7)	1		1,769 (43.5)	2,301 (56.5)	1	0.47	
70–75	406 (17.8)	1,879 (82.2)	0.9 (0.8–1.1)	0.21	1,497 (45.0)	1,830 (55.0)	1.1 (1.0–1.2)		
75–80	360 (17.6)	1,689 (82.4)	0.9 (0.8–1.0)		1,287 (44.7)	1,590 (55.3)	1.1 (1.0–1.2)		0.059
80–85	291 (17.4)	1,382 (82.6)	0.9 (0.7–1.0)		966 (45.2)	1,171 (54.8)	1.1 (1.0–1.2)		
85+	243 (20.0)	972 (80.0)	1.0 (0.8–1.2)		697 (43.5)	904 (56.5)	1.0 (0.9–1.1)		
Townsend deprivation index									
Quintile 1 (least deprived)	302 (15.9)	1,601 (84.1)	1		1,052 (37.3)	1,767 (62.7)	1		
Quintile 2	327 (17.3)	1,559 (82.7)	1.1 (0.9–1.3)	<0.01	1,122 (40.0)	1,682 (60.0)	1.1 (1.0–1.3)	<0.01	<0.01
Quintile 3	352 (18.6)	1,537 (81.4)	1.2 (1.0–1.4)		1,232 (44.2)	1,558 (55.8)	1.3 (1.2–1.5)		
Quintile 4	346 (18.7)	1,506 (81.3)	1.2 (1.0–1.5)		1,295 (46.8)	1,472 (53.2)	1.5 (1.3–1.6)		
Quintile 5 (most deprived)	369 (20.7)	1,410 (79.3)	1.4 (1.2–1.6)		1,496 (53.7)	1,289 (46.3)	2.0 (1.8–2.2)		

^a^Adjusted for other variables in the model.

** Tests for interaction between characteristic and cohort (CFAS I/CFAS II).

Reasons for non-participation were collected in CFAS II only. Just less than half (*n* = 2,944/6,214, 47%) of non-participants were prepared to give a reason for their decision. Not agreeing with surveys in general or not being interested in science (*n* = 987), being too busy (*n* = 776) and not feeling well enough (*n* = 712) accounted for 84% of the reasons not to participate. Of those not prepared to give a reason, 10% (309/3,270) were refusals given by proxy, and 3% (*n* = 87) agreed to be interviewed but were not in when the interviewer arrived.

For CFAS II individuals who were considered too cognitively impaired or too frail upon approach to undertake an interview themselves, most (*n* = 300/378, 79%) were able to provide details of an informant whom they consented for the study team to approach and request information on their behalf (see Table 2). Of the informants approached, ranging from family members and friends to care workers, just under half completed an informant interview (*n* = 146/300, 49%). The stratified random sub-sample generated 1,339 informant interview requests, of which 741 (55%) were successfully undertaken. In around 20% of cases, the respondent refused permission to approach an informant, often feeling they had provided enough information themselves (*n* = 280, 21%). Just over 9% of informants declined the interview (*n* = 127, 9%), and in 191 cases (14%), there was no informant who could be contacted.

**Table 2.** Participation and non-participation in CFAS II informant interviews

	Cambridgeshire (%)	Newcastle (%)	Nottingham (%)	Total (%)
Informant interview				
Requested	78	153	147	378
Completed	26 (33.3)	66 (43.1)	54 (36.7)	146 (38.6)
Refused	37 (47.4)	62 (40.5)	55 (37.4)	154 (40.7)
No informant identified	15 (19.2)	25 (16.3)	38 (25.9	78 (20.6)
Informant interview (random stratified sub-sample)				
Requested	461	462	416	1,339
Completed	301 (65.3)	248 (53.7)	192 (46.2)	741 (55.3)
Respondent refused permission	76 (16.5)	97 (21.0)	107 (25.7)	280 (20.9)
Informant refused interview	38 (8.2)	47 (10.2)	42 (10.1)	127 (9.5)
No suitable informant identified	46 (10.0)	70 (15.2)	75 (18.0)	191 (14.3)

## Discussion

Non-participation among older people increased substantially in the two-decade period between CFAS I and CFAS II. In both cohorts, women and those living in areas of greater deprivation were more likely to be non-participants. Of those approached in both studies, we found no evidence that non-participation was significantly higher in the young-old or the very old.

These findings are consistent with a review of published epidemiological studies [2] that found a decline in participation between 1970 and 2003 that became steeper after 1990, the period between our two studies. Although the reviewers reported a median participation of 74%, this is likely to be an overestimate given the majority of studies reported little or no information on participation. Involvement of general practices remained high in CFAS II, but more decided not to participate than in CFAS I. The challenge of recruitment of general practices to research is well documented, and an issue for many studies [10–12] suggesting increasing pressures on general practice time is the biggest obstacle.

Ascertaining why someone declines to take part in a study is problematic. In CFAS II, just over half of non-participants did not volunteer a reason. A sensitivity analysis found no significant difference in the characteristics of people who provided a reason and those who did not. For those who did, a third said they were not interested in surveys or science despite our attempts to be explicit about the contribution that an individual can make to understanding health and ageing. Anecdotal evidence from study interviewers suggests that some refusals were due to competing requests to participate in research. Although a third provided reasons that suggested it was concerns about their health that discouraged them from participating, over a quarter said they were simply ‘too busy’. While this may be a way of declining participation without causing offence, older people are increasingly likely to be in some form of paid employment [13] and/or be providing unpaid childcare to their grandchildren [14]. With increasing life expectancy, many older people may be caring for surviving parents [15]. With such demands now placed on many older people's time, participation in studies of this kind is perhaps unsurprisingly accorded a lower priority.

Dealing with declining response to study cohort differences has both practical and analytical implications. Our own work comparing prevalence of dementia estimated from CFAS I and CFAS II applied inverse probability weighting to adjust for non-response and conducted a range of sensitivity analyses using extreme scenarios about non-participants [16]. To recruit a cohort of a pre-specified size, a greater number of people need to be approached. This increases the cost of epidemiological studies of older people where recruitment and fieldwork represent the largest proportion of study funding.

Our finding that greater deprivation is associated with non-participation is consistent with other studies [17–21]. It is concerning that those with fewer resources and therefore potentially greater health needs are under-represented in epidemiological evidence. A randomised comparison of opt-out with opt-in study recruitment found greater non-participation and non-response bias with the latter [22], suggesting our own method of recruitment limited further bias.

A review of participation in epidemiological studies found men less likely to participate than women [1], with similar findings from studies specific to older people [21]. In contrast, we found women more likely to be non-participants which may be due to increased caring demands falling disproportionately on older women. We cannot exclude the possibility that living alone is a confounder in the association between female gender and non-participation, but we cannot investigate this without knowledge of the home circumstances of non-participants.

The strength of our analysis is in the ability to directly compare non-participation between two studies identical in design, approach and targeted populations but conducted at different time points. However, we are limited in our comparison to socio-demographic factors, as only this information is available for non-participants. In CFAS I, we have no record of reasons for non-participation and for both ethical reasons and out of courtesy for those we approached, interviewers were only able to ascertain reasons for non-response for those who volunteered this information.

## Conclusion

The findings from our investigation of two epidemiological studies of older people have provided further evidence that non-participation in research is increasing. A decline in the willingness of members of the public to participate in research of this kind poses substantial challenges for researchers at a time when the demand for representative data on ageing populations is high. As communities become more diverse and older people have increasing commitments on their time, innovative approaches are urgently needed to inform study design and recruitment strategies that engage prospective participants.

**Key points**

- Non-participation in research is increasing in epidemiological studies of older people.
- Non-participation is greater among women, those in urban areas, and those living in areas of higher deprivation.
- New ways to engage prospective participants are needed.

## Conflicts of interest

None declared.

## Authors' contributions

L.G. carried out the statistical analysis; E.G. cleaned the data and helped with analysis; L.E.B., L.R. and A.A. had responsibility for fieldwork in Cambridgeshire, Newcastle and Nottingham; C.B. and F.E.M. supervised study design and helped to draft the manuscript. A.A. supervised the analysis and is the guarantor. L.G., E.G. and A.A. wrote the first draft, all authors read and approved the final manuscript.

## Supplementary data

Supplementary data mentioned in the text are available to subscribers in *Age and Ageing* online.

## Funding

This work was supported by the Department of Health; the Medical Research Council; The National Institute of Health Research comprehensive research networks in West Anglia and Trent and the dementias and neurodegenerative disease research networks in Newcastle (grant number G9901400, G0601022). F.E.M. is supported by the MRC U105292687. The funders had no role in the design, implementation, analysis or interpretation of the study.

## Supplementary Material

Supplementary Data
