# Supplementary material for: Changing non-participation in epidemiological studies of older people: evidence from the Cognitive Function and Ageing Study I and II
Source: Age Ageing. 2015 Aug 20;44(5):867–73. doi: 10.1093/ageing/afv101 (PMC4547929; doi:10.1093/ageing/afv101)
Supplement: Supplementary Data [file supp_44_5_867__index.html]

Changing non-participation in epidemiological studies of older people: evidence from the Cognitive Function and Ageing Study I and II — Changing non-participation in epidemiological studies of older people: evidence from the Cognitive Function and Ageing Study I and II — Supplementary Data 

# Changing non-participation in epidemiological studies of older people: evidence from the Cognitive Function and Ageing Study I and II

## Supplementary Data

Supplementary Data

- Supplementary Data - Docx file
