# Supplementary material for: Changing non-participation in epidemiological studies of older people: evidence from the Cognitive Function and Ageing Study I and II
Source: Age Ageing. 2015 Aug 20;44(5):867–73. doi: 10.1093/ageing/afv101 (PMC4547929; doi:10.1093/ageing/afv101)
Supplement: Supplementary Data [file supp_afv101_afv101supp.docx]

**SUPPLEMENTARY DATA**

Webtable 1: Distribution of socio-demographics between people who died before interview and people who were eligible to approach

|  | CFAS I | | CFAS II | |  |
| --- | --- | --- | --- | --- | --- |
| Characteristic | Not Died  N = 9345 (%) | Died  N =542 (%) | | Not Died  N = 14242 (%) | Died  N =1097 (%) |
| Geographical area |  |  | |  |  |
| Cambridgeshire | 3242 (94.5) | 190 (5.5) | | 4277 (/93.3) | 308 (6.7) |
| Newcastle | 3037 (94.2) | 187 (5.8) | | 4950 (93.4) | 355 (6.6) |
| Nottingham | 3066 (94.9) | 165 (5.1) | | 4985 (92.0) | 434 (8.0) |
| Age at sampling date |  |  | |  |  |
| 65 – 70 | 2123 (97.8) | 47 (2.2) | | 4083 (97.8) | 92 (2.2) |
| 70 – 75 | 2285 (97.1) | 68 (2.9) | | 3347 (96.5) | 120 (3.5) |
| 75 – 80 | 2049 (94.8) | 112 (5.2) | | 2915 (93.4) | 206 (6.6) |
| 80 - 85 | 1673 (92.7) | 132 (7.3) | | 2195 (89.4) | 261 (10.6) |
| 85 + | 1215 (87.0) | 182 (13.0) | | 1702 (80.3) | 418 (19.7) |
| Overall median age (IQR) | 75.8 (65.3, 100.8) | 81.6 (65.5,100.5) | | 74.0 (64.0, 100.0) | 82.0 (65.0,104.0) |
| Deprivation |  |  | |  |  |
| Deprivation Quintile 1 | 1883 (94.5) | 110 (5.5) | | 2916 (95.3) | 143 (4.7) |
| Deprivation Quintile 2 | 1856 (94.7) | 104 (5.3) | | 2870 (93.8) | 191 (6.2) |
| Deprivation Quintile 3 | 1861 (94.8) | 102 (5.2) | | 2876 (92.7) | 226 (7.3) |
| Deprivation Quintile 4 | 1870 (95.0) | 99 (5.0) | | 2763 (92.0) | 240 (8.0) |
| Deprivation Quintile 5 | 1839 (93.6) | 126 (6.4) | | 2768 (90.7) | 283 (9.3) |
| Overall median Townsend deprivation score (IQR) | 0.1 (-5.8, 10.5) | 0.2 (-5.9,9.2) | | -0.8 (-6.5, 10.6) | 0.2 (-5.7,9.8) |

Registered patients aged 65 + within geographical area identified by FHSAs / PCTs

Age stratified sample of potential participants drawn

Interview with informant

Interview declined / appointment cancelled / interview terminated

Interviewer visits to arrange interview appointment

Contact study office declining participation

No contact with study office

Study invitation letter sent

Approval given and contact details confirmed

Practice agree to participate

General practices approached

Practice declined to participate

Approval sought from GP to approach participant

GP unwilling for patient to be approached

Interview with participant

Participant unable to complete interview

Informant interview requested (RSS)

Informant interview requested

No informant interview required

Informant interview not obtained

Informant interview not obtained

Interview with informant

*RSS: Random Stratified Subsample

Webfigure 1: Participation identification and recruitment for CFAS I and CFAS II

RSS – Random stratified subsample
